# Can crayfish take the heat? *Procambarus clarkii* show nociceptive behaviour to high temperature stimuli, but not low temperature or chemical stimuli

**DOI:** 10.1242/bio.20149654

**Published:** 2015-03-27

**Authors:** Sakshi Puri, Zen Faulkes

**Affiliations:** Department of Biology, The University of Texas-Pan American, Edinburg, TX 78539, USA

**Keywords:** Nociception, Crayfish, Antenna, *Procambarus clarkii*, Pain

## Abstract

Nociceptors are sensory neurons that are tuned to tissue damage. In many species, nociceptors are often stimulated by noxious extreme temperatures and by chemical agonists that do not damage tissue (e.g., capsaicin and isothiocyanate). We test whether crustaceans have nociceptors by examining nociceptive behaviours and neurophysiological responses to extreme temperatures and potentially nocigenic chemicals. Crayfish (*Procambarus clarkii*) respond quickly and strongly to high temperatures, and neurons in the antenna show increased responses to transient high temperature stimuli. Crayfish showed no difference in behavioural response to low temperature stimuli. Crayfish also showed no significant changes in behaviour when stimulated with capsaicin or isothiocyanate compared to controls, and neurons in the antenna did not change their firing rate following application of capsaicin or isothiocyanate. Noxious high temperatures appear to be a potentially ecologically relevant noxious stimulus for crayfish that can be detected by sensory neurons, which may be specialized nociceptors.

## INTRODUCTION

Many large crustaceans (lobsters, crabs, and crayfish) are cooked as seafood by being boiled alive, which is a controversial practice. Many people believe crustaceans experience pain, while others argue that they do not. Whether an animal experiences pain is complicated, but a much more tractable question is whether a particular species has nociceptors, which are neurons tuned to tissue damage, or to stimuli that could cause tissue damage. It is reasonable to think nociceptors would be widespread across species, since they have obvious and profound survival value (Crook et al., 2014).

Tissue can be damaged in many ways, and accordingly, many nociceptors are polymodal (Kumazawa, 1998; Ashley et al., 2007; Srinivasan et al., 2008), and respond to multiple kinds of stimuli, including temperatures of ∼45°C or more (Defrin et al., 2002; examples in fishes, Ashley et al., 2007; Nordgreen et al., 2009; *Caenorhabditis elegans*, Wittenburg and Baumeister, 1999; Liu et al., 2012; *Hirudo medicinalis*, Pastor et al., 1996, *Drosophila melanogaster*, Tracey et al., 2003; Sokabe et al., 2008), extreme pH, mechanical pressure, and chemicals that often stimulate nociceptors, which are hereafter referred to as nocigenic chemicals. Nocigenic chemicals cause ion channels in nociceptors to open and are usually interpreted as noxious, but the chemicals themselves do not damage tissue. For example, the feeling of extreme heat when eating certain pungent foods is due to chemicals like capsaicin (found in chillies and peppers; Thomas et al., 1998; Tewksbury et al., 2008) or isothiocyanate (found in mustards, horseradish, and wasabi; Sultana et al., 2003b; Sultana et al., 2003a).

As with other sensory stimuli, species vary in what stimuli trigger nociceptors. For example, capsaicin is an agonist for nociceptors in multiple vertebrate (LaMotte et al., 1992; Jordt et al., 2003) and invertebrate (Pastor et al., 1996; Wittenburg and Baumeister, 1999) species. Some vertebrates are insensitive to capsaicin (Szolcsányi et al., 1986; Jordt and Julius, 2002; Park et al., 2008). Fruit flies (*D. melanogaster*) prefer foods with capsaicin, but avoid isothiocyanate (Al-Anzi et al., 2006). These behaviours can also be shaped by experience: in humans, naïve tasters do not prefer pungent foods, but they can be an “acquired taste” (Dib, 1990).

The evolutionary reasons for such variation of response are unknown. While mammalian nociceptors have been studied intensively (Le Bars et al., 2001) because of their relevance for human pain, research on nociceptors in non-mammalian animals, such as fishes (Sneddon, 2002; Sneddon, 2003; Sneddon et al., 2003) and invertebrates (Kavaliers, 1988; Smith and Lewin, 2009; Crook et al., 2011) is less comprehensive. Comparative studies may well create new models for studying nociception and pain.

Arthropods are the most abundant invertebrate taxon, but the suggestion that insects may not have nociceptors persisted into this century (Eisemann et al., 1984; Le Bars et al., 2001). This view is untenable now, given extensive research on nociception in *D. melanogaster* (Tracey et al., 2003; Al-Anzi et al., 2006; Hwang et al., 2007; Tracey, 2007; Sokabe et al., 2008; Neely et al., 2011; Hwang et al., 2012; Kim et al., 2012). There are clear behavioural responses to noxious stimuli (Tracey et al., 2003; Al-Anzi et al., 2006; Chattopadhyay et al., 2012; Johnson and Carder, 2012) occurring in ecologically relevant contexts (Hwang et al., 2007; Johnson and Carder, 2012), and identified genes that are strongly implicated in nociceptors (Tracey et al., 2003; Kim et al., 2012). Nevertheless, such clear evidence for nociceptors does not exist for other species of arthropods, including crustaceans. Insects are descended from crustaceans (Regier et al., 2010), but that insects have nociceptors does not necessarily mean that crustaceans also have nociceptors. For example, some forms of nociception in *D. melanogaster* are mediated by the *painless* gene (Tracey et al., 2003; Al-Anzi et al., 2006), a transient receptor potential A (TRPA) ion channel. Several insect species have four or five TRPA genes, but the crustacean species *Daphnia pulex* has only one TRPA gene (Matsuura et al., 2009), which might not be a *painless* homologue and could have nothing to do with nociception. Nociception may have evolved in insects after the split between insects and crustaceans. Similarly, there has not yet been any physiological identification of crustacean neurons that are tuned to tissue damage.

Molecular and physiological data do not answer whether crustaceans have nociceptors, but, several papers suggest that crustaceans show nociceptive behaviour (Barr et al., 2008; Appel and Elwood, 2009; Elwood and Appel, 2009). Some results of nociceptive behaviour (Barr et al., 2008) have not been replicated in other crustacean species (Puri and Faulkes, 2010). Further, the existing behavioural data can be interpreted in ways that do not require specialized nociceptors to explain the results. For example, several studies have used electric shock as a noxious stimulus to hermit crabs (Appel and Elwood, 2009; Elwood and Appel, 2009) and shore crabs (Magee and Elwood, 2013). Electric shock will activate any electrically excitable cell, including non-neural ones, and the ecological relevance of electric shock is not clear. Injection of formaldehyde is another noxious stimulus (Dyuizen et al., 2012), which is also likely cause non-specific effects on many cells, not just nociceptors, and is a stimulus that a crustacean is unlikely to encounter in the wild. Thus, nociceptive behaviours triggered by such stimuli may represent abnormal responses of the nervous system rather than the workings of a nociceptive sensory system tuned to tissue damage by evolution.

Here, we test whether crayfish respond to noxious temperatures and nocigenic chemicals, using both behavioural and physiological methods. Temperature extremes are ecologically relevant stimuli for crayfish (Payette and McGaw, 2003), and trigger nociceptors in other freshwater species, such as trout (Sneddon et al., 2003). Fruit flies (*D. melanogaster*) respond to nocigenic chemicals, which lead us to hypothesize that crayfish should respond to isothiocyanate, but not capsaicin, provided a homologue to the *D. melanogaster* painless gene was present in crustacean nociceptors. Crustaceans should respond to neither isothiocyanate nor capsaicin if crustaceans have no nociceptors, or nociceptors unlike those described in many other taxa.

Portions of this work have appeared in abstract (Puri and Faulkes, 2009; Puri and Faulkes, 2014).

## MATERIALS AND METHODS

Louisiana red swamp crayfish, *Procambarus clarkii* (Girard, 1852) of both sexes were purchased from a commercial supplier (Carolina Biological Supply Company) and housed individually in small tanks, consistent with previous experiments (Puri and Faulkes, 2010). All experiments complied with U.S. animal welfare regulations.

### Thermal stimuli

#### Behavioural experiments

Because the stimuli used were potentially noxious to the crayfish, all behavioural experiments were conducted so that the stimuli would be brief, and to allow individuals to remove themselves from the noxious stimuli.

**Fig. 1.**
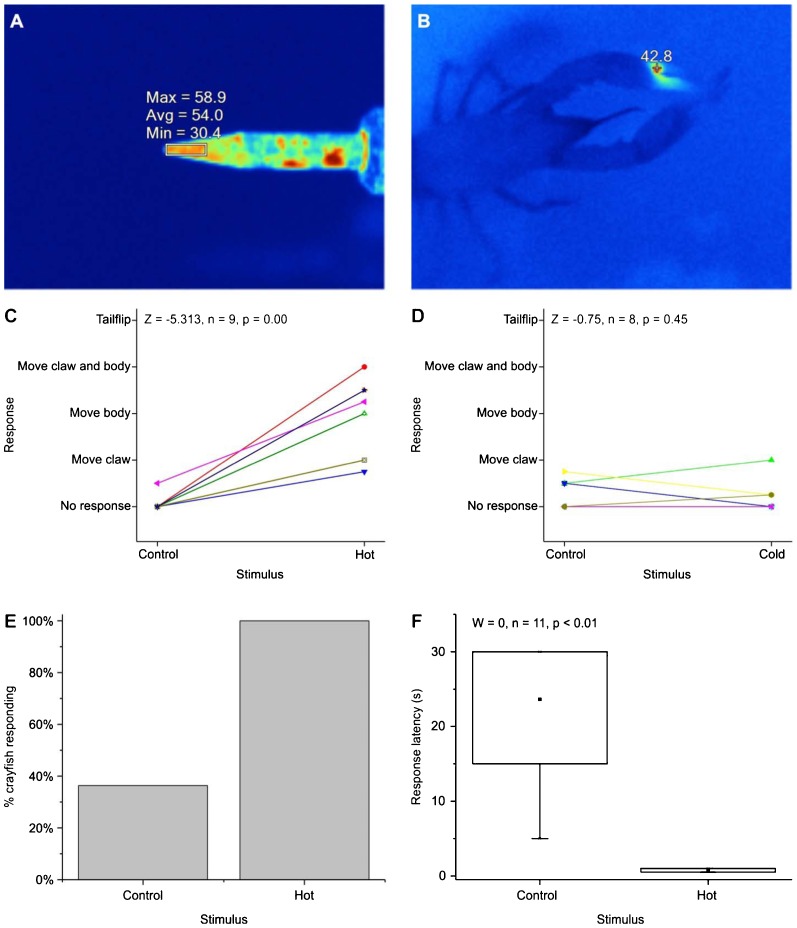
Behaviour of crayfish in response to noxious thermal stimuli. (A,B) Thermal images of (A) soldering iron tip and (B) crayfish immediately after being touched by soldering iron. (C,D) Response of crayfish touched on the claw with (C) high temperature stimulus or (D) low temperature noxious stimulus. (E) Proportion of crayfish responding to touch on claw with high temperature stimulus. n = 11. (F) Latency of response to touch on the antennae. No response within 30 s was coded as 30 s. Dot = mean; line dividing box = median; box = 50% of data; whiskers = 95% of data; asterisks = minimum and maximum. n = 11.

For the first behavioural experiments, crayfish were removed from water and placed in a small tank, and presented with control and high temperature stimuli on the claw. For high temperatures, we touched crayfish with the lightest pressure possible (i.e., touching rather than pressing the tip against the claw) in the nook of the claw (i.e., where the dactyl meets the propus in the interior gripping surface of a chela) with a soldering iron either at ∼20°C (room temperature control) or heated to ∼54°C. The soldering iron was plugged into a variable transformer to reduce its temperature, which was measured with a Fluke Ti9 thermal imaging camera (Fig. 1A). The temperature of the tissue elevated quickly even after a brief touch of the soldering iron (Fig. 1B). If the crayfish did not remove its claw from the soldering iron, we held the soldering iron against the claw for 3 s.

For low temperatures, we touched the nook of the claw with plastic forceps (control), or dry ice (∼−78.5°C) held within the same plastic forceps. Crayfish were touched four times, alternating between the left and right claws, for each condition, and an average was taken of the four responses for analysis. Behaviours were ranked as: 1 = no response, 2 = movement of claw; 3 = movement of body; 4 = movement of claw and body; 5 = tailflip. Latency was measured from the first touch of the control and test stimuli, and latencies longer than 3 s were coded as 3 s.

Because we used antennae for physiological tests, we conducted a second experiment in which we touched crayfish on the antenna with a soldering iron, either at ∼20°C (room temperature control) or heated to ∼54°C. The legs and claws of crayfish were restrained, and crayfish were held in position while the soldering iron was touched to one antenna while the antenna was stationary. We measured whether the crayfish responded to the touch, and the latency of the response to the nearest second. Movement of the antenna within 30 s after the touch was counted as a response. For latency, no response after 30 s was recorded as 30 s. A video camera was placed above the crayfish to record movement of the antenna. Crayfish were given a three hour rest period between the two stimuli.

#### Physiological experiments

Crayfish were anesthetized by chilling on ice, and one second antenna (Sandeman, 1989) was cut at the base, and placed in freshwater crayfish saline (210 NaCl mmol l^−1^, 2.5 KCl mmol l^−1^, 2.5 MgCl_2_ mmol l^−1^, 14 CaCl_2_ mmol l^−1^, and buffered to pH 7.45–7.6 with TRIS; Paul and Mulloney, 1986) at room temperature (∼20°C).

The antenna contains two nerve branches of about equal size. Each branch was teased apart and recorded individually with an extracellular suction electrode. The antenna was held in position by placing pins at the margins of antennae (i.e., not through the tissue).

Stimuli were delivered by 100 µl of physiological saline by a micropipette, either at room temperature or heated to ∼60°C. Because the amount of saline introduced was so small, water temperature in the bath was approximately the same at the beginning and end of the experiment (i.e., room temperature). In no case did the overall bath temperature rise to the levels expected to set off nociceptors (i.e., over 40°C).

Electrical activity was sampled at 20 kHz though a CED 1902 amplifier (Cambridge Electronic Design), HumBug noise filter (Quest Scientific), CED Micro 1401 Mark II analogue-to-digital board (Cambridge Electronic Design), and recorded on a Windows-based PC using Spike 2 version 5.20 software (Cambridge Electronic Design).

The neural activity of the two nerve branches differ slightly in their baseline spontaneous activity and maximum spike size. The branches were sorted into “high-frequency baseline” and “low frequency baseline” by their spontaneous activity, measured by the average spontaneous activity from three individual seconds of recording, selected at random, before the first stimulus was delivered. Only antennae in which baseline recording could be measured from both branches were used in analyses.

Spontaneous activity was monitored throughout the experiment for baseline drift. There was no evidence of consistent changes in baseline activity due to application of the stimulus.

Control and high temperature stimuli were presented in alternating sets: three control stimuli, three high temperature stimuli, followed by two more alternating sets of control and high temperature stimuli (a total of nine control and nine high temperature stimuli each). The response to the stimulus was measured as the total number of action potentials during the 1 s when the stimulus was delivered. To normalize for differences in baseline spontaneous activity, the number of action potentials generated in response to stimuli was divided by the calculated baseline of spontaneous activity. Results were analyzed using Origin 7.5 (OriginLab Corporation).

### Chemical stimuli

#### Behavioural experiments

Two types of behavioural experiments were performed to test for nociceptive responses to nocigenic chemicals: food consumption and antenna swabbing.

Animals were given foods containing capsaicin and isothiocyanate to see if they would avoid these foods. Two separate experiments were conducted using peppers and wasabi rhizomes to test for avoidance of capsaicin and isothiocyanate, respectively.

Anaheim peppers, *Capsicum annuum* L., and habanero peppers, *C. chinense* Jacq., were purchased from a local grocery store. Peppers are pungent due to capsaicin. The pungency of peppers is measured by the Scoville scale (Scoville, 1912), which provides a gross estimation of the capsaicin content. Anaheim peppers rate very low on the Scoville scale, whereas habaneros peppers have high capsaicin content, rating 100,000–500,000 Scoville units (Bosland, 1992; Thomas et al., 1998; Chancellor and de Groat, 1999). One author (Z.F.) ate representative slices of the peppers, similar to those fed to the subjects, to confirm that there were noticeable differences in pungency, and the habanero slices were unpleasantly pungent.

Each crayfish, housed individually, was given two slices of Anaheim pepper and two slices of habanero pepper simultaneously. The amount eaten was checked at 15 minute intervals for 90 min, and then again the following day. The amount eaten was coded as: 1 = not eaten at all, 2 = less than 50% eaten; 3 = 50–89% eaten; 4 = 90–99% eaten; 5 = entirely eaten.

Wasabi rhizomes, *Eutrema japonica* (Miq.) Koidz., were bought from a commercial supplier. There is no equivalent to the Scoville scale for isothiocyanate, and the amount of isothiocyante varies with cultivation practices (Sultana et al., 2002; Sultana et al., 2003b; Sultana et al., 2003a). One author (Z.F.) ate representative slices to confirm that the samples were pungent. Each crayfish was given one sliver of wasabi rhizome. We looked for avoidance of the wasabi rhizome. Similar to the capsaicin experiment, the amount eaten was checked at 15 minute intervals for 90 min and then the following day. The amount eaten was coded as: 1 = not eaten, 2 = less than 25%; 3 = 25–50%; 4 = 51–75%; 5 =  76–99%, 6 = 100%.

In the antenna swabbing experiments, individuals were removed from water and placed on a paper towel, and one antenna was swabbed with either a control (ethanol) or a chemical agonist (10 mmol l^−1^capsaicin in ethanol, or 10 mmol l^−1^ benzyl isothiocyanate in ethanol). The other antenna was not swabbed, so that any changes caused by the mechanical action of swabbing could be detected. Each individual was placed in a tank (175 mm long × 100 mm wide × 90 mm high) filled with ∼50–80 mm of aged tap water. Behaviour was recorded for 10 min using a digital video camera (Logitech). We measured any contact of other portions of the body (i.e., mouth, legs) with either antenna; the movement of the animal in its environment, by recording the number of times the anterior region of the carapace (i.e., eyes) crossed the midline of the tank along its long axis; and tailflips. Ten individuals were tested in each condition. No individuals were tested twice. Following their use in these experiments, animals were kept and housed in the lab. Their status was monitored during routine animal care. Results were analyzed using SPSS 12 for Windows.

#### Physiological experiments

Animals of both sexes were anesthetized by chilling on ice. One second antenna (Sandeman, 1989) was cut and placed in freshwater crayfish saline (210 NaCl mmol l^−1^, 2.5 KCl mmol l^−1^, 2.5 MgCl_2_ mmol l^−1^, 14 CaCl_2_ mmol l^−1^, and buffered to pH 7.45–7.6 with TRIS; Paul and Mulloney, 1986). The nerve was exposed by dissection.

We prepared a dish containing a petroleum jelly well about 10–20 mm in diameter. The antenna was placed across the top of the well, and was then secured with additional petroleum jelly. Crayfish saline was added to the dish outside the well. The well prevented the liquid being tested from interacting with the exposed nerve at the dissected end of the tissue. The nerve tip was placed inside a suction electrode. The recording was allowed to equilibrate for 2 min, which established a baseline. A control liquid (ethanol) was placed in the petroleum jelly well for 1 min. The saline was withdrawn from the well, and the preparation was again allowed to equilibrate for 2 min. Then, the test stimulus (capsaicin or isothiocyanate) was placed in the well for 1 minute. The series of treatments (baseline, control liquid, and test liquid, interleaved with equilibration periods) was conducted at least twice for each individual.

Electrical activity was sampled at 20 kHz though a CED 1902 amplifier (Cambridge Electronic Design), HumBug noise filter (Quest Scientific), CED Micro 1401 Mark II analogue-to-digital board (Cambridge Electronic Design), and recorded on a Windows-based PC using Spike 2 version 5.20 software (Cambridge Electronic Design). The spike sorting capabilities of Spike 2 software were used to identify individual neurons. For each treatment, we made at least five recordings where we were able to distinguish three spikes or more.

## RESULTS

### Crayfish respond to high temperatures but not low temperatures

High, but not low, temperatures caused rapid nociceptive-like behaviours in crayfish (Fig. 1; supplementary material Movie 1). Crayfish consistently responded to touches to both the claw and the antenna with high-temperature soldering iron tip, but less often and less intensely to room temperature control touches. When touched on the claw, every crayfish tested responded more intensely to the high temperature than the control (Fig. 1C). Only 11% of crayfish (1 of 9) responded in less than 2 s when touched with the control, whereas 100% of crayfish responded in less than 2 s when touched with the high temperature stimulus. The behaviours of the crayfish when touched with high temperatures often included repeated tailflipping (an escape response; Wine and Krasne, 1972; Krasne and Wine, 1984; Wine, 1984; Reichert, 1988; Herberholz et al., 2004; Faulkes, 2008), walking rapidly away from the soldering iron, grabbing the soldering iron with the non-touched claw (supplementary material Movie 1).

Crayfish responded to low temperatures touches at the same intensity as control (Fig. 1D). The latency of the response was significantly longer to low-temperature stimuli than controls (Wilcoxon signed-rank test, W = 0, n = 11, p<0.01), which is the opposite pattern predicted if crayfish found the low temperatures noxious and avoided it. In one case, a crayfish grabbed and held on to dry ice for 17 s before releasing it (supplementary material Movie 1).

All crayfish responded to high temperature touches on the antenna by moving the touched antenna away from the soldering iron, but responded to room temperature touches to the antenna less than 40% of the time (Fig. 1E). Crayfish responded to touches of high temperatures in about 1 s (Fig. 1F).

Crayfish showed no long-term damage or changes after either behavioural experiment (e.g., no legs autotomized, loss of function, sudden increase in animal deaths, etc.).

**Fig. 2.**
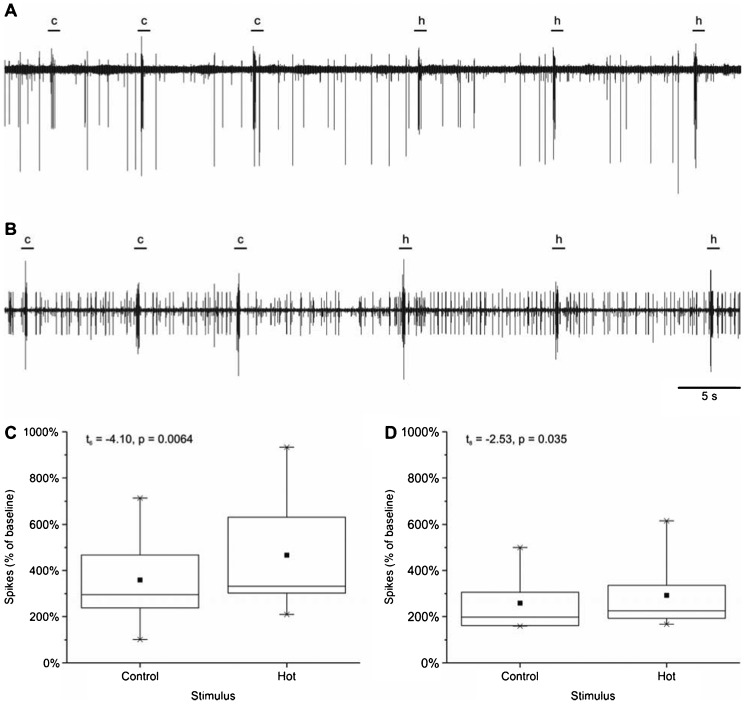
Physiology of neurons in antenna of crayfish in response to high temperature stimuli. (A,B) Representative recordings of low-frequency baseline nerve and high-frequency baseline nerve. Bars above traces indicate approximate delivery of 100 µl saline stimulus; c = control; h = high temperature. (C,D) Increase in number of action potentials relative to baseline spontaneous activity generated in response to delivery of 100 µl of saline in (C) low-frequency baseline nerve, and (D) high-frequency baseline nerve. Dot = mean; line dividing box = median; box = 50% of data; whiskers = 95% of data; asterisks = minimum and maximum.

Neurophysiological recordings in the antennal nerve also showed a difference between high temperature stimuli and room temperature controls, delivered as transient exposure to small amounts of physiological saline. There are two branches of the antennal nerve, which differ in their baseline activity (Fig. 2A,B). Neural activity in the antenna to high temperature stimuli was significantly higher than control stimuli in both the low-baseline (paired t-test, t_6_ = −4.10, p = 0.0064; Fig. 2C) and the high baseline (paired t-test, t_8_ = −2.53, p = 0.035; Fig. 2D) branches of the nerve.

### Crayfish do not respond to nocigenic chemicals

**Fig. 3.**
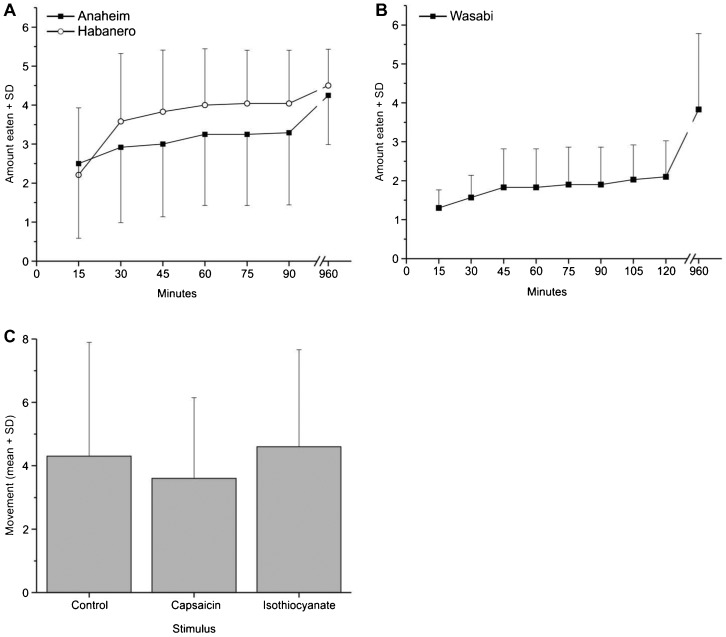
Behaviour of crayfish in response to chemicals that often stimulate nociceptors. (A) Crayfish were presented with peppers containing both low (Anaheim peppers) and high (habanero peppers) concentrations of capsaicin simultaneously. The amount eaten (Y axis) was coded as: 1 = not eaten, 2 = less than 50% eaten; 3 = 50–89% eaten; 4 = 90–99% eaten; 5 = entirely eaten. (B) Crayfish presented with fresh wasabi rhizomes containing isothiocyanate. The amount eaten was coded as: 1 = not eaten, 2 = less than 25%; 3 = 25–50%; 4 = 51–75%; 5 = 76–99%, 6 = 100%. (C) Movement of crayfish measured by number of times individuals crossed between tank halves after application of control (ethanol) or 10 mmol l^−1^ capsaicin 10 mmol l^−1^ isothiocyanate to one antenna. Error bars show standard deviation.

If crayfish detect capsaicin, as many mammals and multiple invertebrate species do, they would be expected to eat the food with low capsaicin concentrations before they ate the food with high capsaicin concentrations. The opposite occurs (Fig. 3A): crayfish ate larger amounts of habanero peppers than Anaheim peppers. Crayfish ate wasabi slices, although they were often slow to do so (Fig. 3B). Wasabi slices tend to float on the surface of the water, making it more difficult for crayfish to grab and handle the food.

Crayfish whose antennae were swabbed with nocigenic chemicals showed no significant differences in activity between animals in the control, isothiocyanate, and capsaicin treatments (ANOVA, F_2,27_ = 0.275, p = 0.762; Fig. 3C). No individuals groomed either antenna, or performed escape tailflips, under any condition.

**Fig. 4.**
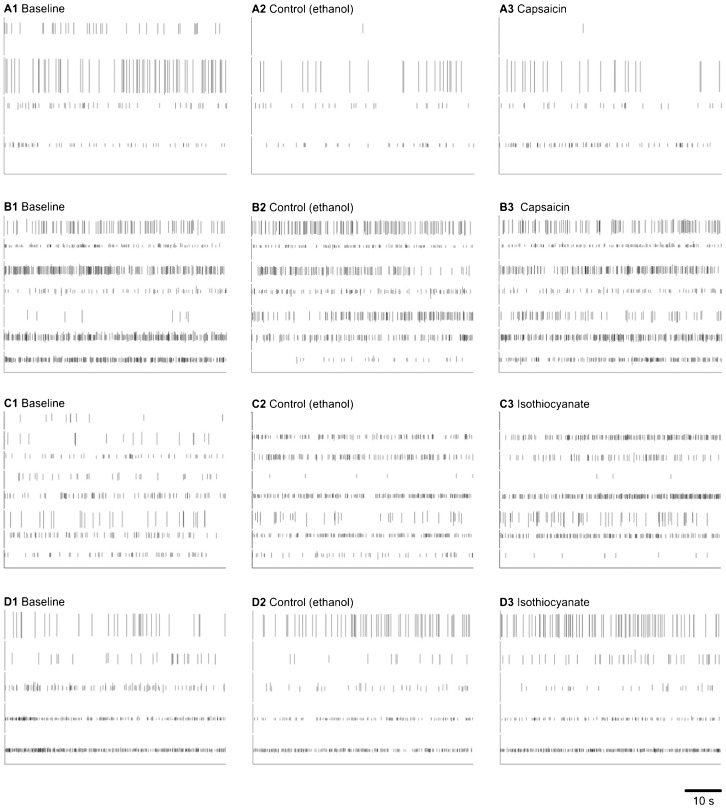
Extracellular recordings of neurons in antenna of crayfish in response to nocigenic chemical stimuli. (A,B) Capsaicin. (C,D) Isothiocyanate. Each row shows recordings from one individual. (1, left column) control with no chemicals showing spontaneous baseline activity; (2, center column) control with ethanol only; (3, right column): test with 10 mmol l^−1^ capsaicin (A,B) or 10 mmol l^−1^ isothiocyanate (C,D) in ethanol.

There was no consistent change in antenna sensory neuron activity when the antennae were presented with either capsaicin (Fig. 3) or isothiocyanate (Fig. 4).

## DISCUSSION

Crayfish respond with nociceptive behaviours to noxious high, but not low, temperatures. Neurophysiological recordings show that antenna neurons can detect short, transient high temperature stimuli, which is consistent with the antenna containing neurons sensitive to high temperatures. Based on the behaviour of the crayfish, we predict that these sensory neurons will be better described as specialized nociceptors rather than generalized thermoreceptors. Previous research showed that crayfish consistently avoid high temperatures, but do not consistently avoid low temperatures (Hall et al., 1978; Payette and McGaw, 2003). This, combined with the results of this study, indicate that high temperatures are noxious stimuli to crayfish with potentially ecological relevance.

We found no evidence that crayfish respond to capsaicin or isothiocyanate, despite multiple tests at different levels of organization. Crayfish did not avoid pungent foods containing capsaicin or isothiocyanate; they did not respond when these chemicals were applied to a major sensory organ (i.e., the antennae); their sensory neurons did not increase their firing rate when exposed to these chemicals. Several hypotheses are consistent with these data. The first hypothesis is that crustaceans do not have nociceptors. The absence of nociceptors seems unlikely, because our experiments with high temperatures show that crayfish have nociceptive behaviour, and there is clear evidence of such neurons in another arthropod, *D. melanogaster* (Tracey et al., 2003; Al-Anzi et al., 2006; Hwang et al., 2007; Tracey, 2007).

The second hypothesis is that crustaceans have nociceptors that are insensitive to both these chemicals. It may be argued that crustacean nociceptors would not respond to capsaicin or isothiocyanate because crustaceans are not ecologically relevant to plants that produce these compounds. The “directed deterrence” hypothesis suggests that nocigenic chemicals like capsaicin have evolved to be noxious to some herbivores (Tewksbury and Nabhan, 2001; Levey et al., 2006). The directed deterrence hypothesis has rarely been tested, although it has been tested for chillies (Levey et al., 2006). The directed deterrence hypothesis does not explain why capsaicin activates nociceptors in organisms for which capsaicin does not appear to be ecologically relevant (e.g., *C. elegans*, Wittenburg and Baumeister, 1999; *H. medicinalis*, Pastor et al., 1996). Capsaicin concentrations in chillies are also correlated to microbial pathogens, which are facilitated by insect feeding (Tewksbury et al., 2008). Based on such ecological interactions, one might predict that insects, which often eat plants, might be sensitive to capsaicin, but *D. melanogaster* is not (Al-Anzi et al., 2006). Whether the nocigenic chemical binds to a nociceptive ion channel in a given species is probably coincidence, although the amount of a nocigenic chemical that a plant produces would be the product of ecological interactions and subsequent natural selection.

We caution against over interpreting these results. Many people are interested in whether crustaceans feel pain because crustaceans are often cooked by boiling them alive. When research on crustacean nociception is presented to the general public, it is often placed in the context of the “lobster in the pot” scenario, even though no previous studies have used high temperature as a noxious stimulus. We wish to be clear that we are not claiming crustaceans generally, or even crayfish specifically, feel pain. We are claiming that crayfish detect and respond to noxious high temperature stimuli in ways that they do not to other potentially noxious stimuli. This suggests that crayfish have nociceptors specialized to detect noxious high temperature stimuli. Nevertheless, whether a species has nociceptors or not is not conclusive evidence that the species feels pain (Varner, 1998), although it is clearly relevant and informs thinking about the question. If crayfish have specialized nociceptors, these sensory neurons may detect other sorts of noxious stimuli, but we have found no other stimuli that elicit these nociceptive behaviours yet.

## Supplementary Material

Supplementary Material

## References

[b1] Al-Anzi B. Tracey W. D. Jr Benzer S. (2006). Response of Drosophila to wasabi is mediated by painless, the fly homolog of mammalian TRPA1/ANKTM1. Curr. Biol. 16, 1034–1040. 16647259 10.1016/j.cub.2006.04.002

[b2] Appel M. Elwood R. W. (2009). Motivational trade-offs and potential pain experience in hermit crabs. Appl. Anim. Behav. Sci. 119, 120–124.

[b3] Ashley P. J. Sneddon L. U. McCrohan C. R. (2007). Nociception in fish: stimulus-response properties of receptors on the head of trout Oncorhynchus mykiss. Brain Res. 1166, 47–54. 17673186 10.1016/j.brainres.2007.07.011

[b4] Barr S. Laming P. R. Dick J. T. A. Elwood R. W. (2008). Nociception or pain in a decapod crustacean? Anim. Behav. 75, 745–751.

[b5] Bosland P. W. (1992). Chiles: a diverse crop. HortTechnology 2, 6–10.

[b6] Chancellor M. B. de Groat W. C. (1999). Intravesical capsaicin and resiniferatoxin therapy: spicing up the ways to treat the overactive bladder. J. Urol. 162, 3–11. 10379728 10.1097/00005392-199907000-00002

[b7] Chattopadhyay A. Gilstrap A. V. Galko M. J. (2012). Local and global methods of assessing thermal nociception in Drosophila larvae. J. Vis. Exp. 2012, e3837. 22643884 10.3791/3837 PMC3466948

[b8] Crook R. J. Dickson K. Hanlon R. T. Walters E. T. (2014). Nociceptive sensitization reduces predation risk. Curr. Biol. 24, 1121–1125. 24814149 10.1016/j.cub.2014.03.043

[b9] Crook R. J. Lewis T. Hanlon R. T. Walters E. T. (2011). Peripheral injury induces long-term sensitization of defensive responses to visual and tactile stimuli in the squid Loligo pealeii, Lesueur 1821. J. Exp. Biol. 214, 3173–3185. 21900465 10.1242/jeb.058131 PMC3168376

[b11] Defrin R. Ohry A. Blumen N. Urca G. (2002). Sensory determinants of thermal pain. Brain 125, 501–510. 11872608 10.1093/brain/awf055

[b12] Dib B. (1990). After two weeks habituation to capsaicinized food, rats prefer this to plain food. Pharmacol. Biochem. Behav. 37, 649–653. 2093168 10.1016/0091-3057(90)90541-o

[b13] Dyuizen I. V. Kotsyuba E. P. Lamash N. E. (2012). Changes in the nitric oxide system in the shore crab Hemigrapsus sanguineus (Crustacea, Decapoda) CNS induced by a nociceptive stimulus. J. Exp. Biol. 215, 2668–2676. 22786644 10.1242/jeb.066845

[b14] Eisemann C. H. Jorgensen W. K. Merritt D. J. Rice M. J. Cribb B. W. Webb P. D. Zalucki M. P. (1984). Do insects feel pain? A biological view. Cell. Mol. Life Sci. 40, 164–167.

[b15] Elwood R. W. Appel M. (2009). Pain experience in hermit crabs? Anim. Behav. 77, 1243–1246.

[b16] Faulkes Z. (2008). Turning loss into opportunity: the key deletion of an escape circuit in decapod crustaceans. Brain Behav. Evol. 72, 251–261. 19001807 10.1159/000171488

[b18] Hall L. W. Jr Cincotta D. A. Stauffer J. R. Jr Hocutt C. H. (1978). Temperature preference of the crayfish Orconectes obscurus. Arch. Environ. Contam. Toxicol. 7, 379–383. 727833 10.1007/BF02332065

[b19] Herberholz J. Sen M. M. Edwards D. H. (2004). Escape behavior and escape circuit activation in juvenile crayfish during prey-predator interactions. J. Exp. Biol. 207, 1855–1863. 15107440 10.1242/jeb.00992

[b20] Hwang R. Y. Zhong L. Xu Y. Johnson T. Zhang F. Deisseroth K. Tracey W. D. Jr (2007). Nociceptive neurons protect Drosophila larvae from parasitoid wasps. Curr. Biol. 17, 2105–2116. 18060782 10.1016/j.cub.2007.11.029 PMC2225350

[b21] Hwang R. Y. Stearns N. A. Tracey W. D. (2012). The ankyrin repeat domain of the TRPA protein painless is important for thermal nociception but not mechanical nociception. PLoS ONE 7, e30090. 22295071 10.1371/journal.pone.0030090 PMC3266251

[b22] Johnson W. A. Carder J. W. (2012). Drosophila nociceptors mediate larval aversion to dry surface environments utilizing both the painless TRP channel and the DEG/ENaC subunit, PPK1. PLoS ONE 7, e32878. 22403719 10.1371/journal.pone.0032878 PMC3293903

[b23] Jordt S.-E. Julius D. (2002). Molecular basis for species-specific sensitivity to “hot” chili peppers. Cell 108, 421–430. 11853675 10.1016/s0092-8674(02)00637-2

[b24] Jordt S.-E. McKemy D. D. Julius D. (2003). Lessons from peppers and peppermint: the molecular logic of thermosensation. Curr. Opin. Neurobiol. 13, 487–492. 12965298 10.1016/s0959-4388(03)00101-6

[b25] Kavaliers M. (1988). Evolutionary and comparative aspects of nociception. Brain Res. Bull. 21, 923–931. 2906273 10.1016/0361-9230(88)90030-5

[b26] Kim S. E. Coste B. Chadha A. Cook B. Patapoutian A. (2012). The role of Drosophila Piezo in mechanical nociception. Nature 483, 209–212. 22343891 10.1038/nature10801 PMC3297676

[b27] Krasne F. B. Wine J. J. (1984). The production of crayfish tailflip escape responses. Neural Mechanisms of Startle Behavior Eaton R C, ed179–211 New York, NY: Plenum Press.

[b28] Kumazawa T. (1998). Primitivism and plasticity of pain – implication of polymodal receptors. Neurosci. Res. 32, 9–31. 9831249 10.1016/s0168-0102(98)00060-1

[b29] LaMotte R. H. Lundberg L. E. Torebjörk H. E. (1992). Pain, hyperalgesia and activity in nociceptive C units in humans after intradermal injection of capsaicin. J. Physiol. 448, 749–764. 1593488 10.1113/jphysiol.1992.sp019068 PMC1176226

[b30] Le Bars D. Gozariu M. Cadden S. W. (2001). Animal models of nociception. Pharmacol. Rev. 53, 597–652. 11734620

[b31] Levey D. J. Tewksbury J. J. Cipollini M. L. Carlo T. A. (2006). A field test of the directed deterrence hypothesis in two species of wild chili. Oecologia 150, 61–68. 16896774 10.1007/s00442-006-0496-y

[b32] Liu S. Schulze E. Baumeister R. (2012). Temperature- and touch-sensitive neurons couple CNG and TRPV channel activities to control heat avoidance in Caenorhabditis elegans. PLoS ONE 7, e32360. 22448218 10.1371/journal.pone.0032360 PMC3308950

[b33] Magee B. Elwood R. W. (2013). Shock avoidance by discrimination learning in the shore crab (Carcinus maenas) is consistent with a key criterion for pain. J. Exp. Biol. 216, 353–358. 23325857 10.1242/jeb.072041

[b34] Matsuura H. Sokabe T. Kohno K. Tominaga M. Kadowaki T. (2009). Evolutionary conservation and changes in insect TRP channels. BMC Evol. Biol. 9, 228. 19740447 10.1186/1471-2148-9-228 PMC2753570

[b35] Neely G. G. Keene A. C. Duchek P. Chang E. C. Wang Q.-P. Aksoy Y. A. Rosenzweig M. Costigan M. Woolf C. J. Garrity P. A. (2011). TrpA1 regulates thermal nociception in *Drosophila.* PLoS ONE 6, e24343. 21909389 10.1371/journal.pone.0024343 PMC3164203

[b36] Nordgreen J. Garner J. P. Janczak A. M. Ranheim B. Muir W. M. Horsberg T. E. (2009). Thermonociception in fish: Effects of two different doses of morphine on thermal threshold and post-test behaviour in goldfish (Carassius auratus). Appl. Anim. Behav. Sci. 119, 101–107.

[b37] Park T. J. Lu Y. Jüttner R. Smith E. S. J. Hu J. Brand A. Wetzel C. Milenkovic N. Erdmann B. Heppenstall P. A. (2008). Selective inflammatory pain insensitivity in the African naked mole-rat (Heterocephalus glaber). PLoS Biol. 6, e13. 18232734 10.1371/journal.pbio.0060013 PMC2214810

[b38] Pastor J. Soria B. Belmonte C. (1996). Properties of the nociceptive neurons of the leech segmental ganglion. J. Neurophysiol. 75, 2268–2279. 8793740 10.1152/jn.1996.75.6.2268

[b39] Paul D. H. Mulloney B. (1986). Intersegmental coordination of swimmeret rhythms in isolated nerve cords of crayfish. J. Comp. Physiol. A 158, 215–224.

[b40] Payette A. L. McGaw I. J. (2003). Thermoregulatory behavior of the crayfish Procambarus clarki in a burrow environment. Comp. Biochem. Physiol. A 136, 539–556. 10.1016/s1095-6433(03)00203-4 14613783

[b41] Puri S. Faulkes Z. (2009). Do crayfish like spicy foods? and other tests of crustacean nociception. Integr. Comp. Biol. 49, e139

[b42] Puri S. Faulkes Z. (2010). Do decapod crustaceans have nociceptors for extreme pH? PLoS ONE 5, e10244. 20422026 10.1371/journal.pone.0010244 PMC2857684

[b44] Puri S. Faulkes Z. (2014). Thermal nociception in Louisiana red swamp crayfish (Procambarus clarkii). Integr. Comp. Biol. 54, e334

[b45] Regier J. C. Shultz J. W. Zwick A. Hussey A. Ball B. Wetzer R. Martin J. W. Cunningham C. W. (2010). Arthropod relationships revealed by phylogenomic analysis of nuclear protein-coding sequences. Nature 463, 1079–1083. 20147900 10.1038/nature08742

[b46] Reichert H. (1988). Control of sequences of movements in crayfish escape behavior. Experientia 44, 395–401.

[b47] Sandeman D. C. (1989). Physical properties, sensory receptors and tactile reflexes of the antenna of the Australian freshwater crayfish Cherax destructor. J. Exp. Biol. 141, 197–217.

[b48] Scoville W. L. (1912). Note on capsicums. J. Am. Pharm. Assoc. 1, 453–454.

[b49] Smith E. S. J. Lewin G. R. (2009). Nociceptors: a phylogenetic view. J. Comp. Physiol. A 195, 1089–1106. 10.1007/s00359-009-0482-z PMC2780683 19830434

[b50] Sneddon L. U. (2002). Anatomical and electrophysiological analysis of the trigeminal nerve in a teleost fish, Oncorhynchus mykiss. Neurosci. Lett. 319, 167–171. 11834319 10.1016/s0304-3940(01)02584-8

[b51] Sneddon L. U. (2003). Trigeminal somatosensory innervation of the head of a teleost fish with particular reference to nociception. Brain Res. 972, 44–52. 12711077 10.1016/s0006-8993(03)02483-1

[b52] Sneddon L. U. Braithwaite V. A. Gentle M. J. (2003). Do fishes have nociceptors? Evidence for the evolution of a vertebrate sensory system. Proc. Biol. Sci. 270, 1115–1121. 12816648 10.1098/rspb.2003.2349 PMC1691351

[b53] Sokabe T. Tsujiuchi S. Kadowaki T. Tominaga M. (2008). Drosophila painless is a Ca^2+^-requiring channel activated by noxious heat. J. Neurosci. 28, 9929–9938. 18829951 10.1523/JNEUROSCI.2757-08.2008 PMC6671277

[b54] Srinivasan J. Durak O. Sternberg P. W. (2008). Evolution of a polymodal sensory response network. BMC Biol. 6, 52. 19077305 10.1186/1741-7007-6-52 PMC2636771

[b55] Sultana T. Savage G. P. McNeil D. L. Porter N. G. Martin R. J. Deo B. (2002). Effects of fertilisation on the allyl isothiocyanate profile of above-ground tissues of New Zealand-grown wasabi. J. Sci. Food Agric. 82, 1477–1482.

[b56] Sultana T. Porter N. G. Savage G. P. McNeil D. L. (2003a). Comparison of isothiocyanate yield from wasabi rhizome tissues grown in soil or water. J. Agric. Food Chem. 51, 3586–3591. 12769529 10.1021/jf021116c

[b57] Sultana T. McNeil D. L. Porter N. G. Savage G. P. (2003b). Investigation of isothiocyanate yield from flowering and non-flowering tissues of wasabi grown in a flooded system. J. Food Compost. Anal. 16, 637–646.

[b58] Szolcsányi J. Sann H. Pierau F.-K. (1986). Nociception in pigeons is not impaired by capsaicin. Pain 27, 247–260. 3797018 10.1016/0304-3959(86)90215-0

[b59] Tewksbury J. J. Nabhan G. P. (2001). Seed dispersal. Directed deterrence by capsaicin in chilies. Nature 412, 403–404. 11473305 10.1038/35086653

[b60] Tewksbury J. J. Reagan K. M. Machnicki N. J. Carlo T. A. Haak D. C. Peñaloza A. L. C. Levey D. J. (2008). Evolutionary ecology of pungency in wild chilies. Proc. Natl. Acad. Sci. USA 105, 11808–11811. 18695236 10.1073/pnas.0802691105 PMC2575311

[b61] Thomas B. V. Schreiber A. A. Weisskopf C. P. (1998). Simple method for quantitation of capsaicinoids in peppers using capillary gas chromatography. J. Agric. Food Chem. 46, 2655–2663.

[b62] Tracey W. D. Jr (2007). Genetics can be painless: molecular genetic analysis of nociception in Drosophila. In *TRP Ion Channel Function in Sensory Transduction and Cellular Signaling Cascades* (ed. W. B. Liedtke and S. Heller), Chapter 16. Boca Raton, FL: Taylor & Francis Group, LLC.

[b63] Tracey W. D. Jr Wilson R. I. Laurent G. Benzer S. (2003). painless, a Drosophila gene essential for nociception. Cell 113, 261–273. 12705873 10.1016/s0092-8674(03)00272-1

[b64] Varner G. E. (1998). In Nature's Interests? Interests, Animal Rights, and Environmental Ethics Oxford: Oxford University Press.

[b65] Wine J. J. (1984). The structural basis of an innate behavioural pattern. J. Exp. Biol. 112, 283–319.

[b66] Wine J. J. Krasne F. B. (1972). The organization of escape behaviour in the crayfish. J. Exp. Biol. 56, 1–18. 21046844 10.1242/jeb.56.1.1

[b67] Wittenburg N. Baumeister R. (1999). Thermal avoidance in Caenorhabditis elegans: an approach to the study of nociception. Proc. Natl. Acad. Sci. USA 96, 10477–10482. 10468634 10.1073/pnas.96.18.10477 PMC17914

