# Supplementary material for: Can crayfish take the heat? Procambarus clarkii show nociceptive behaviour to high temperature stimuli, but not low temperature or chemical stimuli
Source: Biol Open. 2015 Mar 27;4(4):441–8. doi: 10.1242/bio.20149654 (PMC4400587; doi:10.1242/bio.20149654)
Supplement: Supplementary Material [file supp_4_4_441__index.html]

Can crayfish take the heat? Procambarus clarkii show nociceptive behaviour to high temperature stimuli, but not low temperature or chemical stimuli — Can crayfish take the heat? Procambarus clarkii show nociceptive behaviour to high temperature stimuli, but not low temperature or chemical stimuli — Supplementary Material 

# Can crayfish take the heat? *Procambarus clarkii* show nociceptive behaviour to high temperature stimuli, but not low temperature or chemical stimuli

## bio.20149654 Supplementary Material

**Files in this Data Supplement:**

- Supplementary Material - Sakshi Puri and Zen Faulkes doi: 10.1242/bio.20149654
- Movie 1 - **Crayfish responses to noxious thermal stimuli.** Representative examples of responses of crayfish touched with object at room temperature, high temperature (soldering iron), or low temperature (dry ice).
