# Supplementary material for: Can crayfish take the heat? Procambarus clarkii show nociceptive behaviour to high temperature stimuli, but not low temperature or chemical stimuli
Source: Biol Open. 2015 Mar 27;4(4):441–8. doi: 10.1242/bio.20149654 (PMC4400587; doi:10.1242/bio.20149654)
Supplement: Supplementary Material [file supp_bio.20149654_bio.20149654-s1.pdf]

Biology Open (2015) 000, 1–8 doi:10.1242/bio.20149654

Supplementary Material

Sakshi Puri and Zen Faulkes doi: 10.1242/bio.20149654

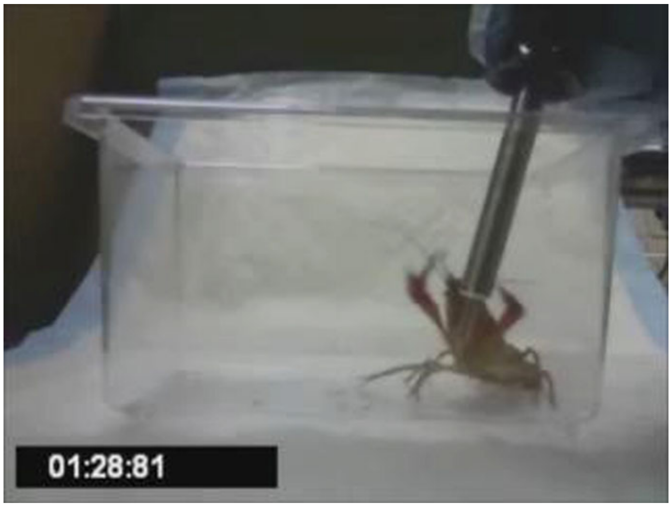

Movie 1. Crayfish responses to noxious thermal stimuli. Representative examples of responses of crayfish touched with object at room temperature, high temperature (soldering iron), or low temperature (dry ice).
